# A Causal Analysis of Life Expectancy at Birth. Evidence from Spain

**DOI:** 10.3390/ijerph16132367

**Published:** 2019-07-03

**Authors:** Pedro Antonio Martín Cervantes, Nuria Rueda López, Salvador Cruz Rambaud

**Affiliations:** Department of Economics and Business, Universidad de Almería, 04120 Almería, Spain

**Keywords:** life expectancy at birth, socioeconomic indicators, panel data, Granger causality test (Dumitrescu-Hurlin)

## Abstract

Background: From a causal point of view, there exists a set of socioeconomic indicators concerning life expectancy. The objective of this paper is to determine the indicators which exhibit a relation of causality with life expectancy at birth. Methods: Our analysis applies the Granger causality test, more specifically its version by Dumitrescu–Hurlin, starting from the information concerning life expectancy at birth and a set of socioeconomic variables corresponding to 17 Spanish regions, throughout the period 2006–2016. To do this, we used the panel data involving the information provided by the Spanish Ministry of Health, Consumer Affairs and Social Welfare (MHCSW) and the National Institute of Statistics (NIS). Results: Per capita income, and the rate of hospital beds, medical staff and nurses Granger-cause the variable “life expectancy at birth”, according to the Granger causality test applied to panel data (Dumitrescu–Hurlin’s version). Conclusions: Life expectancy at birth has become one of the main indicators able to measure the performance of a country’s health system. This analysis facilitates the identification of those factors which exhibit a unidirectional Granger-causality relationship with life expectancy at birth. Therefore, this paper provides useful information for the management of public health resources from the point of view of the maximization of social benefits.

## 1. Introduction

Life expectancy at birth has become an aggregate variable which reflects the influence of a wide variety of indicators (social, economic, environmental, etc.) [1] on the working of modern health systems. On the other hand, the current complex health context, characterized by the constant interrelation of a large number of variables of different types, motivates the causal analysis of these variables which can indicate, among other aspects, the degree to which the resources available to public authorities contribute to the efficient performance of health policies.

A review of the existing literature on this topic (see Section 2) shows that, to the extent of our knowledge, no papers have opted to employ a causal analysis. At this point, it is necessary to clarify that sometimes correlation seems to be confused with causality, which is an obvious error of appreciation which, in statistics, is called the fallacy *cum hoc ergo propter hoc*, that is to say, *a priori*, that correlation does not necessarily imply causality [2]. On other occasions, although the causal analysis is properly contextualized by performing the “classic” Granger causality test for panel data (stacked pairwise Granger causality test), the analysis is not always focused on the variable “life expectancy”, but rather on the causal relationship between GDP *per capita* and health expenditure *per capita* [3]. Therefore, the research question is to address a causal approach to the determinants of health outcome by applying, for the first time in this field, the methodology put forward by Dumitrescu–Hurlin for panel data [4]. This novel procedure is a non-homogeneous causality test, that is to say, a causal empirical analysis which considers the possible correlation of ten socioeconomic variables with the variable “life expectancy at birth” during the period 2006–2016, in the context of the seventeen Spanish regions.

Spain, like Great Britain and Italy, uses a variation of the Beveridge model. In this system, the government provides and finances health care, through taxes. Currently, Spain has a decentralized health system under national coordination. Since 2002, the organization and delivery of health services have been transferred to the seventeen regional health administrations.

This study seeks to examine the causal link between life expectancy at birth and some health care resources and socio-economic factors in the aforementioned Spanish regions from 2006 to 2016. Our contribution is to identify whether the parameters of hospital beds, medical staff in specialized care, medical staff in primary care, nursing staff in specialized care, nursing staff in primary care (all above expressed per 1000 inhabitants), and per capita income are Granger causal for the variable “life expectancy at birth” according to the Granger causality test for panel data (longitudinal, multi-dimensional data involving measurements over time) (Dumitrescu–Hurlin version). This result is of great importance for the design of health policies in our country since, in principle, it highlights the factors which it is necessary to influence in order to increase the level of life expectancy at birth in Spain.

## 2. Literature Review

A review of the empirical literature on this subject reveals the existence of studies which analyze the main determinants of life expectancy using a global approach and macroeconomic data. This type of research analyzes the total effect of the use of health care on the health status of citizens and thereby indicates the implications of different economic policies. Focusing our attention on works which follow a macro approach as referred to developed countries, we classify the determinants of the health status of the population in three categories, namely socio-economic factors, health resources, and lifestyle-related factors. With respect to the socio-economic factors, almost all works consider gross domestic product or per capita income [5,6,7], and with less frequency some indicator of income distribution [8,9,10]. Unemployment is also analyzed [11,12,13] as a determinant of the health status of the population as a representative measure of the fluctuations of the economic cycle. Other relevant macroeconomic variables used in this type of analysis are inflation [14] and gross capital [15].

Education is frequently included in these analyses [16,17,18] as well as the level of employment [5,6,19]. Among demographic variables, some works include the age of the population, i.e., the percentage of those over 60 or 65 [20,21].

Within the category of socioeconomic factors, some works include variables of an institutional nature such as the type of health system [22,23] or the level of fiscal decentralization, corruption and political rights [24]. Another factor which has been incorporated into this type of analysis is pollution, generally represented by emissions of polluting substances [2,6,15,19,25] or by some environmental quality indicator [26]. Recently, existing literature has been enriched by introducing globalization [27] and some indicators of social development [28] as possible determinants of health status.

Regarding health resources, it is common to use some expression of health expenditure. In this way, we can consider either the total expenditure [21,29,30], the entire component of public health expenditure [6,9,19,31], or public and private health expenditure separately [32]. Other authors have incorporated other items such as expenditure on pharmaceutical products [33,34,35] and various categories of other social expenditure in order to assess their differential effect on health status [36,37,38]. Part of this literature also incorporated some proxy variables of health resources (mainly doctors and nurses [19,29]) and, less frequently, the number of beds [31,38]. Additionally, medical innovation [39] (measured by the use of innovative medicines) and the requirement of having a medical prescription for the consumption of certain medications [40] have been analyzed as conditioning factors of the health status.

In order to identify the effects of health habits and lifestyle, it is usual in this type of literature to include other specific variables such as the consumption of tobacco and alcohol, and others concerning diet, such as the intake of vegetables, fruits, sugar, butter, calories, fats, proteins, or even the level of obesity. Within this group of works, we can highlight the papers by [29,30,31,33,34,41].

In Spain, we can find some works on the determinants of the health status from a microeconomic point of view [42]. On the other hand, a regional setting has been employed in other papers but dealing with the efficiency of the Spanish health system [43,44,45]. However, our paper occupies an intermediate position with respect to the former studies, since our aim is to analyze the determinants of the Spanish health system by using macro data of Spanish regions.

In addition, [46] investigate at a regional level and by using aggregate data, the effect of decentralization on health care outcomes in Spain by testing whether a greater decentralization is linked to improvements in population health between 1992 and 2003. To do this, they use infant mortality and life expectancy as dependent variables. They find that income, decentralization, and health care resources have an important influence on both infant mortality and life expectancy. In the same line, [47] analyzes the relationship between unemployment and mortality by using data from some Spanish provinces during the period 1980–1997.

## 3. Materials and Methods

One of the extensions of the well-known Granger causality test [48] is its possible application to panel data starting from a fixed effects model [49] (known as the stacked pairwise Granger causality test):

(1) $$Y_{i,t}=\alpha +\sum_{k=1}^{K}\beta_{ik}X_{t-k}+\sum_{k=1}^{K}\gamma_{ik}Y_{t-k}+\varepsilon_{i,t}$$

where $X_{i,t}$ and $Y_{i,t}$ are the observations of two stationary variables corresponding to an individual *i* in a period *t* and with a number of delays *K* exactly the same for all individuals. The null hypothesis in this model is:

(2) $$H_{0}:\gamma_{i1}=\gamma_{i2}=\cdots =\gamma_{iK}=0,$$

which states the equality of coefficients corresponding to the *N* individuals included in the panel, that is to say, derived from all cross-sectional observations.

However, the previous way of conceptualizing Granger’s causality, restricted to panel data, has some weaknesses related to the viability of such a test [50] which depends on the selected number of items *N* within a given time horizon *T*. [51,52], or its adequacy or, more correctly, the functionality (or working schema) of the null hypothesis (2) since, when considering that a variable $X_{t}$ causes another $Y_{t}$ in a data panel, it is assumed that a null hypothesis has been added to that already proposed [53].

To overcome these drawbacks, Dumitrescu and Hurlin [4] designed a homogeneous non-causality test, derived from (1), in which a *balanced panel* composed of $X_{i,t}$ and $Y_{i,t}$, that is to say, by observing two stationary variables corresponding to an individual *i* in a period *t* and with a number of delays *K* exactly the same for all individuals, it is possible to violate the “classical” assumption (heterogeneous causality test) by assuming that coefficients $\gamma_{ik}$, being invariant with respect to time, can differ among individuals, thus leading to a new null and alternative hypothesis:

(3) $$\mathrm{Homogeneous}\;\mathrm{non}\text{-}\mathrm{causality}\;\mathrm{test}\;\left\{ \begin{array}{l} H_{0}:\gamma_{i1}=\gamma_{i2}=\cdots =\gamma_{iK}=0;\;i=1,\ldots ,N\\ H_{1}:\left\{ \begin{array}{l} \gamma_{i1}=\gamma_{i2}=\cdots =\gamma_{iK}=0;\;i=1,\ldots ,N_{1}\\ \gamma_{i1}\neq 0\;\mathrm{or}\;\ldots \;\mathrm{or}\;\gamma_{iK}\neq 0;\;i=N_{1}+1,\ldots ,N \end{array}\right. \end{array}\right.$$

It should be observed that the alternative hypothesis establishes the plausible causality between the variables $X_{it}$ and $Y_{it}$ for some individuals, but not for all of them, so the rejection of $H_{0}$ does not necessarily imply the existence of causality among some individuals [54]. This hypothesis is known as the hypothesis of homogeneous non-causality and differs from (2) in that the alternative hypothesis allows the causality between X and Y for some individuals, but not for all.

Nevertheless, the contrast of hypothesis (3) is formulated under the assumption that, in the case of a causal relationship given a set of *N* items, the vectors $\gamma_{i1}$ must be strictly identical when adding, as an alternative hypothesis, the existence of $N_{1}$ ($N_{1}=1,2,\ldots ,N-1$) items over which there is no *a priori* explicit causal relationship. Dumitrescu and Hurlin [4] resolve these drawbacks by determining the standard regressions of the Granger causality for each individual in order to obtain the individual Wald statistics and, starting from there, calculate the statistic $\bar{W}$ (average Wald or *W*-bar):

(4) $$\bar{W}=W_{N,t}^{HNC}=\frac{1}{N}\sum_{i=1}^{N}W_{i,t},$$

where $W_{i,t}$ is the Wald statistic applied to the *i*-th individual in *t*, which corresponds with the null hypothesis $H_{0}:\gamma_{i,t}=0$. Analogously, taking into account the hypothesis that the calculated Wald statistics are independent and identically distributed for each of the individuals, it can be demonstrated that the standardized statistics follow a standard normal distribution, when *T* and *N* tend to infinity and *K* denotes the selected number of lags:

(5) $$\bar{Z}=(\bar{W}-K)\sqrt{\frac{N}{2K}}\underset{T,N\to \infty }{\overset{d}{\to }}N(0,1).$$

Moreover, given a fixed dimension of *T*, with T>5+3K, the estimated standardized statistic $\tilde{Z}$ also follows a standard normal distribution:

(6) $$\tilde{Z}=\frac{T-3K-3}{T-3K-1}(\bar{W}-K)\sqrt{\frac{N}{2K}\frac{T-3K-5}{T-2K-3}}\underset{N\to \infty }{\overset{d}{\to }}N(0,1).$$

This last aspect determines the number of delays or lags which must be selected for the Dumitrescu and Hurlin homogeneous non-causality test [12], although originally these authors did not specify a given or optimal number of delays [54], whereby a viable solution may be the combined use of estimators derived from the information criteria by Akaike or Hannan-Quinn, and the Bayesian analysis. However, in our particular study, we have implemented only one time-lag because we were constrained by T>5+3K in order that the Wald statistics could be independently distributed with finite second order moments. In other words, since we are using a time horizon of 11 years, *K* had to be one taking into account the aforementioned restriction (condition that, given the database used in our work, would be violated with two or more lags).

Finally, the contrast of the null hypothesis in (3) is made according to the values of $\bar{Z}$ and $\tilde{Z}$, by comparing them with the standard critical values, rejecting the null hypothesis and therefore concluding that there is causality in the Granger sense according to the procedure of Dumitrescu and Hurlin [4] when $\bar{Z}$ or $\tilde{Z}$ are greater than the standard critical values, taking into account that, for panel data in which *N* and *T* are relatively large, it is more appropriate to test the null hypothesis by means of $\bar{Z}$, leaving the use of $\tilde{Z}$ for those cases where *N* is relatively large and *T* is relatively small.

Given the aforementioned defined variables, we are going to propose the following model in which the subscripts *i* and *t* denote, respectively, the different Spanish regions and the period corresponding to the analyzed time horizon (2006–2016):

(7) $$\begin{array}{c} \mathrm{LEAB}=\beta_{0}+\beta_{1}{\mathrm{BEDS}}_{it}+\beta_{2}{\_\mathrm{NUR}\mathrm{SPE}}_{it}+\beta_{3}{\mathrm{NUR}\_\mathrm{PRI}}_{it}+\beta_{4}{\mathrm{CHRON}}_{it}+\beta_{5}{\mathrm{MED}\_\mathrm{SPE}}_{it}+\\ \beta_{6}{\mathrm{MED}\_\mathrm{PRI}}_{it}+\beta_{7}{\mathrm{STUDI}}_{it}+\beta_{8}{\mathrm{POVER}}_{it}+\beta_{9}{\mathrm{PC}\_\mathrm{INCOM}}_{it}+\beta_{10}{\mathrm{SENES}}_{it}+\varepsilon_{it}, \end{array}$$

where i=1, 2,…,N, t=1, 2,…,T and where $\varepsilon_{i,t}$ represents the random perturbation of the model ($\varepsilon_{i,t}\approx N(0,\sigma^{2})$).

Instead of carrying out a prototypical econometric analysis focused on the unit root test, the cointegration or the estimation of the long-term coefficients of the model previously stated (i.e., through the Dynamic Ordinary Least Squares (D-OLS) and Fully Modified Ordinary Least Squares (F-MOLS) procedures), we are going to focus on the analysis of stationarity of the time series included in the panel data and on the implementation of the Granger causality test by Dumitrescu–Hurlin for panel data. With respect to stationarity, this analysis is prescriptive given that, in the Granger causality test, the analyzed time series must be stationary by definition. At this point, it is necessary to indicate that there is no single statistical test which automatically corroborates the stationarity of the series. In order to draw different conclusions based on whether or not these tests show relatively similar results, more than one stationarity test is usually applied. In this way, we have only applied the Im–Pesaran–Shin (IPS) test, taking advantage of its simplicity and its relatively easy interpretation.

However, among all existing stationarity tests for panel data such as Levin–Lin–Chu (LLC test), Maddala–Wu (MW test), Breitung (test B) and IPS test [55], we have opted for the latter because it leads to the elimination of the serial correlation of the analyzed variables, having a large capacity in relatively small samples [56] such as the one used in this paper.

On the other hand, the IPS test is an ad hoc stationarity test for the implementation of the procedure by Dumitrescu–Hurlin, given that it considers the heterogeneity between the different cross sections. An important characteristic of this test is that it facilitates that the values of $\rho_{i}$ (autoregressive coefficients) vary indistinctly between the sections in the panel, by allowing some series, but not necessarily all of them, to be stationary.

The hypothesis contrast of the IPS test is formulated as follows:

(8) $$\mathrm{IPS}\;\mathrm{test}\;\left\{ \begin{array}{l} H_{0}:\rho_{i}=0;\;i=1,\ldots ,N\\ H_{1}:\left\{ \begin{array}{l} \rho_{i}<0;\;i=1,\ldots ,N_{1}\\ \rho_{i}=0;\;i=N_{1}+1,\ldots ,N \end{array}\right. \end{array}\right.$$

from where it can be derived that the null hypothesis represents the existence of a unitary root, meaning no presence of stationarity in data. This hypothesis will be rejected (considering that the series is stationary in at least one cross-section of the panel), by comparing the value of the statistic *t* and the probabilistic critical values provided by the IPS test.

Finally, it is necessary to emphasize that, although this test has recognized a bidirectional contrast in which the variable “life expectancy” could act both as cause and effect, we opted for a unidirectional contrast in which this variable is the exclusive cause of the different explanatory variables listed above. The bidirectionality, in this specific case, would be completely illogical.

With respect to the methodology proposed in this paper, we have tried to look for an ad hoc implementation of the causality in Granger by taking into account the nature of the data used (panel data). In order to contextualize this research, it is convenient to indicate how the causality in Granger represents an analytical framework which has evolved since its original definition [48] expressly focused on time series and linear causality until multiple revisions and readjustments.

**Figure 1:**
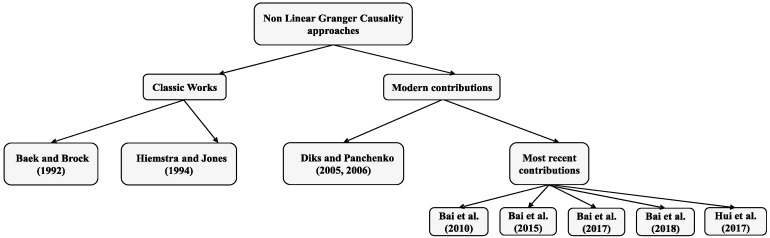
Non-linear Granger causality approaches summary. Source: Own elaboration.

One of the revisions of the scheme originally drawn up by [48] starts from inferring that the plausible causal relationship between two variables, if any, would hardly be strictly linear in nature [57]. The adequacy of Granger causality in the field of non-linearity is one of the big fields in which research has been diversified, showing the Figure 1 a brief summary of the most notable works focused on non-linear Granger causality:

Properly speaking, the non-linear causality analysis of Granger began with [58], and was subsequently readjusted by [59], a transcendental contribution in this field which for more than ten years would guide most of non-linear causal analyses in which both causal approaches, linear and non-linear relationship (in the sense of Granger) between the price and trading volumes of the New York Stock Exchange (NYSE) studied. The fundamental scheme of the nonlinear causality of [59], on which most of subsequent investigations would be based, is focused on the determination of a central limit theorem (CLT) of its statistic test by implementing the asymptotical properties of multivariate U-statistics.

[60] demonstrate, by using Monte Carlo simulations, that the lack of consistency is the main failure in the estimators used in the procedure designed by [59], by proposing, alternatively, a new non-parametric causal test and including a series of practical guidelines for nonparametric Granger causality testing. Consequently, [61] design an extension of the Diks and Panchenko test [60] which is applied to the US grain market. This test is characterized because it applies the data-sharpening as a bias reduction method.

[62] also choose to revise the non-linear test of [59], by suggesting new estimators of the probabilities in order to improve its asymptotic properties in accordance with the original procedure included in [59]. In the same way, [63] also review the non-linear test by [59] by constructing a novel nonlinear causality test in multivariate settings. [64] present a test contemplating different lags of variables and [65], taking [59] as reference, recalculate the probabilities and rebuild the CLT of the new statistic test, by concluding how their estimates are more consistent than the one shown in [59].

Finally, [66] create a completely original methodology to quickly, efficiently, and effectively analyze the nonlinear causal relationship given a group of variables, based on the particularity (also the advantage) that it is not required as a rule to know the exact nonlinear features and the detailed non-linear forms of the variable $Y_{t}$.

Obviously, once the non-linear field of causality in Granger was described in a very brief way, we would have to highlight three works whose practical implementation could greatly support our study: [65,66,67], especially if they are compared and put into perspective with the results obtained by the Dumitrescu–Hurlin procedure. However, if these non-linear causality approaches have not been applied in this work, it is simply due to reasons of operative nature, derived from the little data which compose our database.

In the first case [64], it would be quite difficult to employ a variable number of lags whilst in the following two papers [65,66] we presume that, for the same reasons, the results were not as robust and consistent as they should be, not because of any weakness of these causal models of proven efficacy (see, for example, the application of [66] in the SP index), but to the small size of the database used. In effect, in our opinion, any of these three works would be of great importance as a new methodological perspective in the causal analysis of life expectancy, opting for the nonlinear perspective, always more representative than the strictly linear one (see [57]).

## 4. Data

In our analysis, we have used panel data for the period 2006–2016 referred to life expectancy at birth and another ten socioeconomic variables closely related to it, the description of which is presented in Table 1. Thus, a database has been configured composed of 3179 observations, that is to say, 17 annual observations of the dependent and independent variables for each of the 17 Spanish regions.

The selection of these variables has been made based on the existing literature on this topic, which shows some consensus in considering health resources, socioeconomic factors, and lifestyle as determinants of health status. Unfortunately, in this work it has not been possible to include some variables representative of lifestyle, such as the percentage of smokers or drinkers, among others, as such information for each Spanish region is not available for the analyzed period. This lack of availability for the analyzed period also explains that the territorialized health expenditure has not been included. However, other health resources which are financed by this type of expenditure, namely personnel (doctors and nurses), or materials (hospital beds) have been considered.

Table 2 shows the summary of descriptive statistics of variables included in Table 1.

## 5. Results

Table 3 shows the result of the IPS test of the different variables included in this analysis, in levels and first differences, by considering a single delay of the variables and under the assumption that, in the test equation, both the individual intercept and the trend of each series are included. It can be observed that, for most analyzed variables, the null hypothesis is rejected, which supports the property of stationarity in levels and first differences, except for a small number of variables indicated with an asterisk.

**Figure 2:**
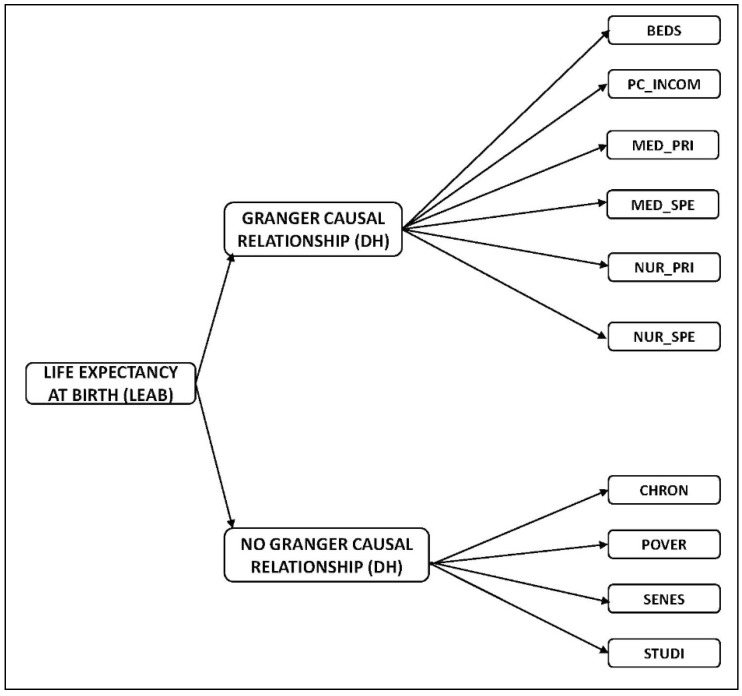
Causal relationships resulting from the causality test for panel data (Dumitrescu–Hurlin version). Source: Own elaboration.

Concerning the contrast of the Granger causality for panel data according to the Dumitrescu–Hurlin procedure, Table 4 shows the Wbar and Zbar statistics, as well as the critical values of this test according to the null hypothesis (3), by stating the causal relationships outlined in Figure 2.

## 6. Discussion

This research has analyzed the socioeconomic indicators which have exhibited a relation of causality with the life expectancy at birth in Spanish regions. Our findings have concluded that, according to the Granger causality test for panel data (Dumitrescu–Hurlin version), the explanatory variables hospital beds, medical staff in specialized care, medical staff in primary care, nursing staff in specialized care, nursing staff in primary care (all above expressed per 1000 inhabitants), and per capita income cause the variable “life expectancy at birth”. This result is very important for the design of health policies in Spain as it identifies the main factors to which it is necessary to give attention in order to increase the life expectancy at birth.

This study is aimed at contributing to the progress of research on health status determinants. Part of existing literature has included resources in physical terms such as the number of physicians and, to a lesser extent, the availability of nurses. An advantage of our analysis is that we have incorporated both personal resources to overcome this restriction in part of the empirical evidence since nurses also occupy an important place in the provision of health care.

Our results confirm previous researches since the number of doctors was found to be significant in improving health outcomes [2,29,31]. Specifically, our findings coincide with previous studies carried out in Spain which explore the determinants of life expectancy, in particular the contribution by [46] which differs from our approach in the following items:

1. [46] also incorporate infant mortality as a dependent variable.
2. These scholars also use fiscal decentralization as an explanatory variable.
3. Finally, our paper is more exhaustive as it incorporates new independent variables such as people with long-term disease or health problems, poverty rate, level of studies, and senescence.

With respect to the methodology employed, our perspective is causal whilst [46] use panel data with fixed vs. random effects. Additionally, logarithms have been applied over the two dependent variables and real *per capita* income and the number of general practitioners.

More recently, some authors have suggested that health care development (measured by input indicators such as the number of hospital beds and the number of doctors per 1000 inhabitants, among others) could significantly improve life expectancy at birth [28]. However, other researchers have concluded that longevity is not explained by the amount of health care provisions, such as the number of hospital beds and the number of health care staff [38]. In addition, a previous study has revealed that, despite its (spurious) positive correlation, the availability of medical specialists has a low impact on mortality rates, in comparison with the economic and social variables which have been used as control variables [71]. On the other hand, in this type of literature, the impact of hospital beds in high-income countries is more ambiguous, and can be explained by the development of high-tech health care provisions which reduce the average length of hospitalization [7].

Whereas the role played by health care resources is more debated in existing literature, there is some consensus in concluding that socioeconomic and lifestyle factors [7] are important determinants of the population’s health status. One of the major socioeconomic factors which has been considered in the literature of life expectancy, is income. At this point, our results are consistent with previous studies showing the importance of income in improving life expectancy at birth [18,72,73,74].

## 7. Conclusions

The main contribution of this paper is threefold. On the one hand, it is the first time that the Granger causality test (Dumitrescu–Hurlin version) has been applied to a causal analysis within the health sector. This contribution becomes more relevant if we take into account the few studies which analyze the causality of health outcome in a country [2]. In this sense, subsequent contributions partially confirm our results in a study carried out in Turkey (using data covering the period 1975–2015), where causality relationships were analyzed (according to Granger) between *per capita* health expenditure, the number of doctors per thousand inhabitants and life expectancy in different age segments, and also between their variations [75]. In this way, it is necessary to repeat that the presence of a correlation between two variables does not necessarily imply the existence of causality. Thus, a corroborated conclusion in the literature is the existence of a close relationship between medical consultation rates and life expectancy as the highest consultation rates are presented in countries with the highest life expectancy [76]. However, this simple correlation does not necessarily imply causality, since the totality of living standards can influence consultation rates and life expectancy.

Second, we contribute to the analysis of life expectancy from a regional perspective. In this sense, it is evident that some studies based on macro data to identify the determinants of health status, have been generalized at country level. However, those works carried out at state, regional or municipal level are less frequent, such as those referred to North American states [5,10,77], the provinces of Canada [29,35], Spain [47], and China [28]; and to Brazilian municipalities [78].

Third, we must point out that another added value of this paper is the use of panel data, which is essential when dealing with information based on different Spanish regions.

Most of the previous empirical research on the determinants of health status have considered some parameter of health care expenditure. One limitation of our work is that we have not included this monetary resource because it is not available for Spanish regions for the entire period 2006–2016. The main restriction of this paper lies in the limited number of available observations, which has resulted in a reduction of the number of delays to one. Despite this limitation, our research analysis provides a relevant contribution to the evaluation of the impact on health and a useful tool for its possible implementation in public health programs.

Finally and as future research, we intend to investigate this type of contrast by using a database with longer time horizon and by incorporating new possible explanatory factors of life expectancy at birth.

## Figures and Tables

**Table 1:** Description of variables and their sources. **Source**: Own elaboration.

Variable	Short Definition	Source
LEAB	Life expectancy at Barth	Key indicators of the National Health System [67]
CHRON	People with long-term disease or health problems (population percentage greater than or equal to 16 years)	Survey of Living Conditions [68]
PC_INCOM	Average net annual income per person and unit of consumption by autonomous communities (euros)	Survey of Living Conditions [68]
POVER	Poverty rate (%)	Key indicators of the National Health System [67]
STUDI	Level of studies: Population from 16 to 64 years with 2nd level of secondary and post-secondary, non-higher education (%)	Labor Force Statistics [69]
SENES	Senescence: Population percentage greater than or equal to 65 years	Statistics of Continuous Register [70]
MED_SPE	Medical staff in specialized care per 1000 inhabitants	Key indicators of the National Health System [67]
MED_PRI	Medical staff in primary care per 1000 inhabitants	Key indicators of the National Health System [67]
NUR_SPE	Nursing staff in specialized care per 1000 inhabitants	Key indicators of the National Health System [67]
NUR_PRI	Nursing staff in primary care per 1000 inhabitants	Key indicators of the National Health System [67]
BEDS	Hospital beds per 1000 inhabitants	Key indicators of the National Health System [67]

**Table 2:** Variables and summary statistics. **Source**: Own elaboration.

Variables											
	LEAB	BEDS	NUR_SPE	NUR_PRI	CHRON	MED_SPE	MED_PRI	STUDI	POVER	PC_INCOM	SENES
Mean	82.46	3.24	3.25	0.66	29.26	1.84	0.78	23.70	20.13	10,542.05	17.74
Median	82.49	3.30	3.16	0.66	28.80	1.79	0.76	23.70	18.10	10,493.00	17.80
Maximum	85.22	4.35	4.93	0.89	45.90	2.55	1.10	29.70	40.20	14,838.00	24.10
Minimum	79.77	2.17	2.53	0.47	16.10	1.37	0.59	16.20	5.30	6668.00	12.10
Std.Dev.	1.12	0.49	0.50	0.10	5.87	0.23	0.10	2.56	7.73	1880.22	2.90
Skewness	−0.19	−0.49	1.02	0.36	0.22	0.62	1.37	−0.34	0.44	0.27	0.22
Kurtosis	2.67	2.64	3.56	2.58	2.84	2.90	5.60	3.06	2.23	2.24	2.25
Jarque-Bera test	1.93	8.52	34.55	5.32	1.69	12.22	111.04	3.55	10.70	6.81	5.89

**Table 3:** Stationarity test by Im–Pesaran–Shim for the analyzed sample. Source: Own elaboration.

IPS Test Null Hypothesis: Unitary Root (Assumes the Process of Unitary Roots at an Individual Level)				
**A. Levels**				
**Series**	***t*** **-Statistic**	**Probability**	**Cross Sections**	**Observations**
BEDS (*)	1.58573	0.9436	17	153
NUR_SPE	0.65073	0.7424	17	153
NUR_PRI	−2.00706	0.0224	17	153
CHRON	−0.49536	0.3102	17	153
LEAB (*)	1.02956	0.8484	17	153
MED_SPE	0.19513	0.5774	17	153
MED_PRI	−1.91310	0.0279	17	153
STUDI	−2.00483	0.0225	17	153
POVER	0.37674	0.6468	17	153
PC_INCOM	−4.19584	0.0000	17	153
SENES	−0.83526	0.2018	17	153
**B. First Differences**				
**Series**	***t*** **-Statistic**	**Probability**	**Cross Sections**	**Observations**
BEDS	−0.56214	0.287	17	136
NUR_SPE	−0.80952	0.2091	17	136
NUR_PRI	−1.50039	0.0668	17	136
CHRON	0.03717	0.5148	17	136
LEAB	−3.75752	0.0001	17	136
MED_SPE	−0.60668	0.272	17	136
MED_PRI	−1.2299	0.1094	17	136
STUDI	−1.4124	0.0789	17	136
POVER	−1.98178	0.0238	17	136
PC_INCOM (*)	1.9624	0.9187	17	136
SENES	0.60758	0.7283	17	136

(*) means that, for these variables, the null hypothesis is not rejected.

**Table 4:** Causality test for panel data (Dumitrescu–Hurlin version) for the analyzed sample. Source: Own elaboration.

Null Hypothesis	Wbar Statistic	Zbar Statistic	Probability
BED does not homogeneously cause LEAB	1.92798	0.77748	0.4369
NUR_SPE does not homogeneously cause LEAB	2.22473	1.21445	0.2246
NUR_PRI does not homogeneously cause LEAB	2.00144	0.88564	0.3758
CHRON does not homogeneously cause LEAB	0.71378	−1.01049	0.3123
MED_SPE does not homogeneously cause LEAB	3.31763	2.82378	0.0047
MED_PRI does not homogeneously cause LEAB	2.40673	1.48244	0.1382
STUDI does not homogeneously cause LEAB	1.12455	−0.40561	0.685
POVER does not homogeneously cause LEAB	0.92645	−0.69733	0.4856
PC_INCOM does not homogeneously cause LEAB	2.7489	1.98631	0.047
SENES does not homogeneously cause LEAB	0.50179	−1.32265	0.186
